# PD-1 inhibitor toripalimab with gemcitabine as a neoadjuvant therapy for muscle-invasive bladder urothelial carcinoma: A case report

**DOI:** 10.1097/MD.0000000000028591

**Published:** 2022-01-14

**Authors:** Huaqi Yin, Ma Yongkang, Guan Bao, Zhao Shiming, He Chaohong, Yang Tiejun

**Affiliations:** Department of Urology, The Affiliated Cancer Hospital of Zhengzhou University, Henan Cancer Hospital, Zhengzhou, China.

**Keywords:** programmed cell death protein 1, toripalimab, urothlial carcinoma

## Abstract

**Rationale:**

Routine neoadjuvant therapy for muscle-invasive bladder urothelial carcinoma prior to radical surgery is curative. With the increase in cancer immunotherapy, neoadjuvant immunotherapy has been used as an important complement to neoadjuvant chemotherapy for muscle-invasive urothelial carcinoma. Toripalimab is a recombinant, humanized IgG4 monoclonal antibody directed against programmed cell death protein 1 and received the first global approval for the treatment of unresectable or metastatic melanoma in China on December 17, 2018.

**Patient concerns and diagnosis:**

A 57-year-old man was admitted to our hospital because of hematuria for 1 week. The patient was diagnosed pathologically with muscle-invasive bladder urothelial carcinoma.

**Interventions and outcomes:**

The patient received neoadjuvant toripalimab combined with gemcitabine therapy. The patient showed partial response. Subsequently, radical cystectomy was performed.

**Lessons:**

Toripalimab combined with gemcitabine exhibited accurate antitumor activity and may be a promising novel neoadjuvant therapy for muscle-invasive urothelial carcinoma.

## 1 Introduction

Bladder cancer (BC) is one of the most common urinary malignancies, accounting for approximately 90% of all urothelial carcinomas. At diagnosis, approximately 25% of patients with BC have muscle-invasive or metastatic disease.^[1]^ The standard treatment for muscle-invasive bladder urothelial carcinoma is radical cystectomy, often accompanied by adjuvant or neoadjuvant chemotherapy. However, approximately 50% of patients with muscle-invasive BC (MIBC) have a poor prognosis owing to recurrence or metastasis, which is partly caused by disseminated tumor cells after radical cystectomy.^[2–4]^ Neoadjuvant therapy could theoretically reduce the risk of cancer relapse or metastasis by diminishing the tumor burden, degrading the tumor stage, and increasing the tumor resection rate. Therefore, seeking effective neoadjuvant therapy for MIBC will be beneficial for improving prognosis.

Toripalimab, developed by Shanghai Junshi Bioscience Co., Ltd (Junshi Bio), is a recombinant, humanized IgG4 monoclonal antibody directed against programmed cell death protein 1 (PD-1) and received the first global approval for the treatment of unresectable or metastatic melanoma in China on December 17, 2018.^[5]^ In a phase II clinical study (NCT03013101), the objective response rate of patients with advanced melanoma treated with toripalimab was 20.7%, 38.5%, and 11.9% in the overall population and programmed cell death protein ligand 1 (PD-L1) positive and PD-L1 negative subgroups, respectively.^[5,6]^ Many clinical trials evaluating the curative effects of toripalimav alone or in combination with chemotherapy or targeted therapy in various cancers are ongoing. In a case report, a patient with pulmonary sarcomatoid carcinoma progression benefitted from toripalimab combined with local radiotherapy.^[7]^ Toripalimab, lenvatinib, and hepatic arterial infusion chemotherapy for advanced HCC resulted in an objective response rate of 59.2%, which was higher than that of lenvatinib alone (9.3%).^[8]^ The approved dosage of toripalimab in melanoma is 3 mg/kg every 2 weeks, which was also confirmed to be tolerant to urothelial cancers.^[9]^ In many settings, toripalimab is used as a second-or third-line therapy. In this case, we report the successful application of toripalimab combined with gemcitabine as neoadjuvant therapy for MIBC.

## 2 Case presentation

**Figure 1:**
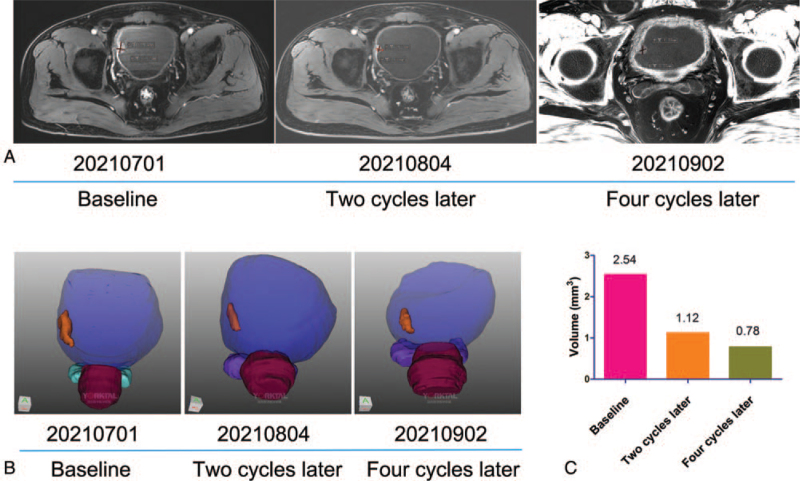
Time line of treatments of the patient and changes in MR scan and MRI 3-dimensional image reconstruction.

A 57-year-old man was admitted to our hospital with hematuria for 1 week on July 1, 2021. Dynamic contrast-enhanced pelvic magnetic resonance imaging (MRI, 20210701) revealed an occupying lesion of 18.24 mm × 13.37 mm (Fig. 1A left) involving the right bladder wall without positive inguinal lymph nodes. MRI 3-dimensional image reconstruction was also used to assess the volume of the lesion, which exhibited a mass of 2.54 mm^3^ (Fig. 1B left and C). Bone scans, chest computed tomography, and abdominal ultrasound showed no positive findings. Cystoscopy verified the neoplasm, and a pathological biopsy demonstrated high-grade urothelial carcinoma. Thus, the patient was diagnosed with muscle-invasive bladder urothelial carcinoma. Considering that deep muscle invasion may increase the risk of tumor dissemination, we planned to administer neoadjuvant therapy before radical cystectomy. We decided to administer toripalimab plus gemcitabine as neoadjuvant therapy after obtaining patient consent.

Neoadjuvant toripalimab plus gemcitabine was divided into 2 stages, each of which contained 2 cycles (1 cycle every 2 weeks). In each cycle, 3 mg/kg toripalimab and 20 mg/kg gemcitabine were infused intravenously infusions every 2 weeks. After 2 cycles of treatment, the efficiency of the combination therapy was assessed. If a complete or partial response was observed, the therapy was continued until the last cycle. Otherwise, the therapy was terminated. Radical cystectomy was performed after termination of the combination therapy.

Neoadjuvant toripalimab plus gemcitabine was administered to the patient on July 5, 2021. After 2 cycles of treatment, the antitumor efficiency was assessed. MRI scan (20210804) showed a significant shrink of the lesion of 9.9 mm × 7.57 mm (Fig. 1A middle) and the volume in MRI-3D image reconstruction was 1.12 mm^3^ (Fig. 1B middle and C). The patient was then treated with 2 other cycles of toripalimab plus gemcitabine on August 5 and 18, 2021. Based on the MRI on September 2, 2021, the lesion (9.55 mm × 6.88 mm) did not vary significantly (Fig. 1A right). However, MRI-3D image reconstruction still revealed a reduction of the tumor with a volume of 0.78 mm^3^ (Fig. 1B middle and C). Collectively, the patient showed a significant partial response to neoadjuvant toripalimab plus gemcitabine. On September 6, 2021, radical cystectomy was performed.

No serious adverse events were observed during the neoadjuvant therapy. In the first period, the patient experienced local dermatitis that disappeared 2 days later. In the second cycle of treatment, the patient experienced mild nausea that remitted without treatment.

Taken together, neoadjuvant toripalimab plus gemcitabine showed promising efficacy and safety in MIBC patients.

## 3 Discussion

Muscle-invasive bladder urothelial cancer, which easily relapses and metastasizes, often requires multimodal therapeutic approaches that include neoadjuvant therapy, surgery, and adjuvant therapy. With the improvement of radical cystectomy and pelvic lymph node dissection, positive surgical margins and lymph nodes were no more than fear. However, many patients with MIBC still experience recurrence and metastasis, which may be caused by disseminated tumor cells during or after surgery.^[10]^ Therefore, reducing the activity of tumor cells and decreasing tumor cell dissemination helps reduce metastatic recurrence and improve the prognosis of MIBC.

Neoadjuvant cisplatin-based chemotherapy is routinely recommended to improve oncological outcomes in patients with MIBC. Although most patients could benefit from neoadjuvant cisplatin-based chemotherapy, some patients, especially the elderly, were ineligible for this approach owing to severe toxicity. Therefore, new neoadjuvant therapies for MIBC are required.

As with the in-depth study of tumor immunology, immunotherapy is a promising treatment approach for a wide range of cancers. Cancer immunotherapy aims to repair or reactivate an impaired immune system during the development and progression of tumors. Immune checkpoint inhibitors, the most common type of cancer immunotherapy, have been successfully used to treat various cancers, including urothelial carcinoma.^[11]^ PD-1, expressed on the surface of multiple immune cells, binds to its ligand, PD-L1, which is expressed by tumor cells and results in the formation of an inhibitory tumor immune microenvironment that facilitates tumor progression. Targeting PD-1/PD-L1 has shown considerable benefits in various malignancies, including urothelial carcinoma. In the adjuvant setting, pembrolizumab targeting PD-1 has been approved as the first-line treatment for patients with urothelial carcinoma who are cisplatin-ineligible and induces a robust and durable response in these populations.^[12]^ In a phase 3 clinical trial, patients with muscle-invasive urothelial carcinoma who received adjuvant nivolumab had longer disease-free survival than those received placebo.^[13]^ In neoadjuvant settings, immunotherapy for MIBC has been evaluated in several ongoing clinical trials. Ipilimumab, an anti-CTLA-4 antibody, was the first neoadjuvant immunotherapy for MIBC and showed considerable antitumor effects.^[14]^ Several PD-1/PD-L1 inhibitors have been used in neoadjuvant clinical trials. In addition, the combination of several immune checkpoint inhibitors has shown reduced outcomes for urothelial carcinoma. Therefore, the development of cancer immunotherapy could provide more options for urothelial carcinoma.

Toripalimab is a recombinant, humanized IgG4 monoclonal PD-1 antibody that was developed by Shanghai Junshi Bioscience Co., Ltd. (Junshi Bio) and was first approved for the treatment of unresectable or metastatic melanoma in China on December 17, 2018. Subsequently, the safety and efficacy of toripalimab have been verified in various solid tumors, and the combination of toripalimab with other therapeutic strategies has been undergoing clinical trials worldwide.^[15]^

In this case, a patient with muscle-invasive bladder urothelial carcinoma was treated with toripalimab plus gemcitabine before radical cystectomy. The volume of the solid tumor decreased by 70% with no severe adverse effects. The decrease in the solid tumor volume and clinical staging make tumor resection easier and may also reduce the risk of tumor cell dissemination during or after surgery. This successful application demonstrated the potential role of neoadjuvant toripalimab combined with gemcitabine in the treatment of muscle-invasive bladder urothelial carcinoma.

## 4 Conclusion

In this case, toripalimab combined with gemcitabine exhibited accurate antitumor activity and may be a promising novel neoadjuvant therapy for muscle-invasive urothelial carcinoma.

## Acknowledgments

We thank the Pathology Department of Henan Cancer Hospital.

## Author contributions

**Conceptualization:** Zhao Shiming.

**Data curation:** Huaqi Yin.

**Formal analysis:** Ma Yongkang.

**Investigation:** Guan Bao.

**Methodology:** Yang Tiejun.

**Project administration:** Guan Bao.

**Resources:** Ma Yongkang.

**Supervision:** He Chaohong, Yang Tiejun.

**Writing – original draft:** Huaqi Yin.

**Writing – review & editing:** Yang Tiejun.
